# The effect of preoperative hospital anxiety and depression scale evaluation on the clinical outcomes following posterior lumbar interbody fusion

**DOI:** 10.1016/j.bas.2026.107483

**Published:** 2026-10-17

**Authors:** Luc W.F. van Haaster, Joep Kitzen, Gijsbert M. Overdevest, Willem Pondaag, Godard C.W. de Ruiter, Carmen L.A. Vleggeert-Lankamp

**Affiliations:** a Department of Neurosurgery, Leiden University Medical Center, Albinusdreef 2, Leiden, 2333 ZA, the Netherlands; b Department of Orthopedics, Maasstad Hospital, Maasstadweg 21, Rotterdam, 3079 DZ, the Netherlands; c Department of Neurosurgery, HAGA Hospital, Els Borst-Eilersplein 275, The Hague, 2545 AA, the Netherlands; d Department of Neurosurgery, Spaarne Gasthuis Hospital, Spaarnepoort 1, Hoofddorp, 2134 TM, the Netherlands; e Department of Neurosurgery, Alrijne Hospital, Simon Smitweg 1, Leiderdorp, 2353 GA, the Netherlands; f Department of Neurosurgery, Haaglanden Medical Center, Lijnbaan 32, The Hague, 2512 VA, the Netherlands

**Keywords:** Hospital anxiety and depression scale, Mental status, Posterior lumbar interbody fusion

## Abstract

**Introduction:**

Preoperative anxiety and depression may influence outcomes in patients undergoing posterior lumbar interbody fusion (PLIF).

**Research question:**

Do elevated preoperative anxiety and/or depression affect postoperative patient-reported outcome measures (PROMs) one year after PLIF?

**Material and methods:**

Patients from an observational cohort study on smoking and PLIF, who completed one-year follow-up were included. Preoperative and one-year postoperative Hospital Anxiety and Depression Scale (HADS), Oswestry Disability Index (ODI), Numeric Rating Scale (NRS) for back- and leg pain, Five Dimensions Health Questionnaire (EQ-5D), Visual Analogue Scale (VAS) health scores and Perceived Recovery scores were assessed. Outcomes were compared between patients with normal (<13) and abnormal (≥13) HADS scores and between anxiety (HADS-A) and depression (HADS-D) subgroups.

**Results:**

Eighty-seven patients were included with a mean age of 59 years. Forty patients had normal and 47 abnormal baseline HADS scores. Patients with abnormal HADS scores reported significantly worse preoperative PROMs but achieved comparable one-year improvements. Despite comparable change scores, they continued to report worse NRS back pain, EQ-5D, HADS, and perceived recovery at one year. In the HADS-A subgroup, significant postoperative differences were observed for NRS back pain and perceived recovery. In the HADS-D subgroup, ODI, NRS back pain, and EQ-5D remained significantly worse. The proportion of patients achieving the minimum clinically important difference was similar across groups.

**Discussion and conclusion:**

PLIF provides meaningful functional improvement in patients with elevated preoperative anxiety and depression. However, these patients may experience lower postoperative status and satisfaction despite comparable improvements, highlighting the importance of preoperative patient education.

## Highlights

• Abnormal HADS scores were associated with worse baseline PROMs.
• Patients with abnormal HADS scores achieved similar postoperative gains in PROMs.
• Poorer pain, quality of life and recovery persisted at one year.
• Depression showed stronger associations with outcomes than anxiety.
• Preoperative HADS assessment may improve patient counseling.

## 1 Introduction

Posterior lumbar interbody fusion (PLIF) is a common surgical technique for the treatment of degenerative lumbar conditions, including spondylolisthesis with accompanying spinal stenosis, recurrent disc herniation, and foraminal stenosis (Mobbs et al., 2015; Spiker et al., 2019). Managing patients’ expectations is important for patient satisfaction. Patients with high expectations preoperatively are less satisfied with surgical outcomes than patients with moderate or low expectations, despite having similar postoperative functional outcomes following lumbar fusion surgery (Zhang et al., 2024). The larger the discrepancy is between patient expectations and postoperative outcomes following spine surgery, the lower the patient satisfaction (Witiw et al., 2018). Predicting postoperative outcomes has therefore received increasing attention in recent years, as it may help optimize preoperative counseling and align patient expectations with realistic surgical results.

Mental health status, particularly preoperative symptoms of depression and anxiety, is increasingly recognized as a clinically relevant prognostic factor in spinal surgery. These symptoms are common among patients undergoing lumbar procedures, although estimates vary substantially by population and instrument. The prevalence of preoperative depression among these patients ranges from 18% to 45% while anxiety symptoms are reported in 19% to 50% of this population (Rahman et al., 2021; Held et al., 2022; Chen et al., 2024). Mental status can be evaluated using the Hospital Anxiety and Depression Scale (HADS), a validated instrument designed to assess core symptoms of anxiety and depression without taking physical symptoms into account. The questionnaire comprises fourteen items, divided into two subscales: an anxiety scale and a depression scale, each consisting of seven items (Zigmond and Snaith, 1983). The effect of mental health status has been evaluated in heterogeneous cohorts of patients undergoing various spinal procedures, including decompression and/or fusion, in which depression was associated with more symptoms and poorer functional outcomes at 12- and 24 months postoperatively (Held et al., 2022; Carreon et al., 2020). This aligns with a 2024 meta-analysis demonstrating that patients with depression undergoing lumbar spine surgery presented with greater disease severity both pre- and postoperatively compared to those without depression, while nonetheless achieving clinically meaningful improvements in disability (Javeed et al., 2024). Similarly, a 2024 registry-based study of more than 45,000 patients undergoing lumbar spine surgery found that those with comorbid anxiety and depression reported lower patient satisfaction, greater disability, and higher levels of both back and leg pain postoperatively. Moreover, this population demonstrated higher readmission rates, increased rates of surgical site infection, and a greater need for revision surgery (Deshpande et al., 2024). A key limitation shared by these studies is the inclusion of heterogeneous patient cohorts, which may introduce confounding, as the procedures examined vary considerably in their indications, degree of invasiveness, and expected postoperative recovery trajectories.

Focusing on a single surgical technique reduces procedural heterogeneity and may limit the risk of confounding. However, to date, this relationship has not been specifically examined in a homogenous cohort consisting exclusively of patients undergoing PLIF surgery.

Therefore, the aim of this study is to evaluate the association between preoperative anxiety and depression scores and postoperative treatment outcomes in patients undergoing PLIF surgery, using the Oswestry Disability Index (ODI), Numeric Rating Scale (NRS) for back pain and leg pain, EuroQol Five Dimensions Health Questionnaire (EQ-5D), Visual Analogue Scale (VAS) health and Perceived Recovery as outcome measures.

## 2 Materials and methods

### 2.1 Patient selection

This study presents a secondary analysis of data from an observational cohort study. Therefore, sample size calculations were not indicated for this study. Patients were selected from the Smoking Cessation trial, an ongoing Dutch prospective multicenter study evaluating the effects of both preoperative and postoperative smoking and smoking cessation on the outcomes of posterior lumbar interbody fusion surgery. The original study protocol was approved by the Medical Ethical Committee of Leiden The Hague Delft (NL83858.058.23). All participants of the Smoking Cessation trial provided written informed consent. The subset of patients included in the present study had additionally consented, within the original informed consent form, to the use of their data for future research purposes.

For the present study, a subgroup was included consisting of all consecutive patients from the six participating centers that completed the 12-months clinical follow-up period between June 2023 and April 2026. All patients aged between 18 and 75 years old and eligible for posterior lumbar interbody fusion (L1-S1) due to degenerative causes were eligible for inclusion. Exclusion criteria were revision surgery, malignancy, fractures following trauma, complex deformities, and pregnancy.

Baseline questionnaires were collected, consisting of the following items: Oswestry Disability Index (ODI), Numeric Rating Scale (NRS) for backpain and leg pain, Hospital Anxiety Depression Scale (HADS), EuroQol Five Dimensions Health Questionnaire (EQ-5D) and Visual Analogue Scale (VAS) health. At 2, 6 and 12 months postoperatively, the same questionnaires were obtained, in addition to the Perceived Recovery score. Results from previous assessments were not visible to the patient.

The demographic data collected at baseline included age, gender, Body Mass Index (BMI), diagnosis, surgical level, and smoking status.

### 2.2 Hospital Anxiety Depression Scale (HADS)

The HADS is a scale that quantifies the anxiety and depression experienced by a patient. It is a validated scale consisting of seven depression items and seven anxiety items. Each item is scored on a scale from 0 to 3, where 0 means ‘not applicable to me at all’ and 3 means ‘very applicable to me’. Scores range from 0 to 21 on both scales, with a higher score indicating more anxiety and/or depression (Zigmond and Snaith, 1983). The HADS has been validated across diverse clinical populations, with acceptable to good internal consistency (Cronbach's α 0.70-0.90 for both subscales) and moderate to strong correlations with other established measures of anxiety and depression (e.g. Beck Depression Inventory, State-Trait Anxiety Inventory) (Olssøn et al., 2005; Bjelland et al., 2002). In addition, the scale shows good discriminant validity and responsiveness to clinical change (Herrmann, 1997).

For this study, a preoperative total HADS score of <13 was classified as normal, while a score of ≥13 was considered abnormal. It should be acknowledged that the literature reports considerable variation in the cut-off values used for the total HADS score, with some studies applying thresholds of ≥18 (Bjelland et al., 2002). For this study, a score of ≥13 was chosen as a more conservative cut-off. When examining the anxiety and depression subscales separately, a HADS-Anxiety score <8 was regarded as normal, whereas a score of ≥8 was categorized as abnormal, in accordance with the optimal cut-off values established by Bjelland et al. (2002). The same threshold was applied to the HADS-Depression subscale. All thresholds were selected prior to data analysis.

### 2.3 Oswestry Disability Index (ODI)

The ODI was used to grade a patient's functionality regarding back and leg function. This validated questionnaire consists of ten items related to the impact on pain, daily function, and well-being. Each item is rated on a scale from 0 to 5. The total score ranges from 0, representing no disability and distress, to 50, indicating maximum disability and loss of function, which was converted to a 0-100% score (Daltroy et al., 1996).

### 2.4 Numeric Rating Scale (NRS) for pain

The pain experienced by patients was measured using a score they assigned to their perceived pain. Patients were asked about their NRS leg pain and NRS back pain. Pain was assessed as ‘average over the past week prior to the follow-up moment’. Pain intensity was scored on a scale of 1 to 10, where 1 meant ‘no pain at all’ and 10 meant ‘the worst pain imaginable’ (Chiarotto et al., 2019).

### 2.5 EuroQol Five Dimensions Health Questionnaire (EQ-5D)

The EQ-5D measures five dimensions related to quality of life: mobility, self-care, usual activities, pain/discomfort, and anxiety/depression. These dimensions are measured on a three-point scale. For each patient's health state, a utility score was calculated representing the value generally assigned to that state (Rabin et al., 2001).

### 2.6 VAS health

Patients rated their perceived health state on a scale from 0, representing a state as bad as death, to 100, indicating optimal health (Rabin et al., 2001).

### 2.7 Perceived Recovery

Perceived recovery was assessed using a 7-point Likert scale, with 1 representing complete recovery, and 7 indicating a state worse than ever. This scale is considered valid and responsive to change (Bombardier, 2000).

### 2.8 Statistical analysis

Statistical analysis were conducted using IBM SPSS Statistics Version 31. Demographic data were presented by means and standard deviations when appropriate. Independent samples t-tests were used to compare continuous variables between groups. Chi-square tests were applied to assess associations between categorical variables. Fisher's exact test was used when expected cell counts were less than five. The correlation between baseline HADS and pre- and postoperative PROMS and one-year change scores was assessed with Spearman correlation tests. A p-value of less than 0.05 was considered statistically significant. All statistical methods and outcome measures were pre-specified prior to data analysis.

## 3 Results

A total of 87 patients were included with a mean age of 59.38 ± 9.80 years and BMI of 27.67 ± 4.17 from six participating hospitals: Maasstad Hospital (n = 34), Spaarne Gasthuis Hospital (n = 22), Alrijne Hospital (n = 15), Haaglanden Medical Centre (n = 13), Leiden University Medical Centre (n = 2) and HAGA Hospital (n = 1).

Forty patients had a normal HADS score at baseline, whereas 47 patients had an abnormal HADS score. Baseline demographics were comparable between the two groups, while all preoperative PROMS were consistently worse in patients with an abnormal HADS score, only lacking significance for the NRS back pain score (p = 0.133). One year postoperatively, patients with an abnormal HADS score at baseline scored worse than the patients with a normal HADS score on all outcome parameters and this difference was significant for NRS back pain (p = 0.027), EQ-5D (p = 0.022) and perceived recovery scores (p = 0.008), but not for ODI (p = 0.072), NRS leg pain (p = 0.282) and VAS health (p = 0.150). The change in value per parameter was additionally evaluated, and this revealed that the one-year changes in ODI, NRS leg, EQ-5D and VAS health scores were greater in the abnormal HADS group, compared to the normal HADS group, though not significantly. However, in evaluating the proportion of patients achieving the minimal clinically important difference (MCID), the percentage of patients that reached the MCID for the ODI score was significantly higher for patients with abnormal HADS scores (p = 0.024). On the contrary, the proportion of patients achieving satisfactory perceived recovery scores was higher for patients with normal HADS (p = 0.033) (Table 1).

**Table 1:** Overview of study participants demographic data, differentiated by the HADS score. Abbreviations: HADS; Hospital Anxiety and Depression Score, BMI; Body Mass Index, ODI; Oswestry Disability Index, NRS; Numeric Rating Scale, EQ-5D; EuroQol Five Dimensions Health Questionnaire, VAS; Visual Analogue Scale, MCID; Minimal Clinically Important Difference. *Perceived recovery assessed one year postoperatively.

	HADS Category		
	Normal (HADS <13)	Abnormal (HADS ≥13)	P-value

N	40	47	
Male, n (%)	20 (50.0%)	19 (40.4%)	0.371
Smoker, n (%)	8 (20.0%)	12 (25.5%)	0.541

	Mean (SD)	Mean (SD)	

Age (years)	59.45 (10.68)	59.32 (9.11)	0.951
BMI	27.45 (3.92)	27.86 (4.40)	0.643
Preoperative			
ODI	32.39 (15.44)	46.57 (16.06)	**<0.001**
NRS leg	6.30 (1.90)	7.15 (1.90)	**0.041**
NRS back	6.00 (2.15)	6.68 (2.03)	0.133
EQ-5D	0.55 (0.26)	0.40 (0.30)	**0.018**
VAS health	66.18 (16.93)	55.47 (21.65)	**0.013**
HADS	8.00 (2.82)	19.17 (5.97)	**<0.001**

One year postoperative			
ODI	14.86 (18.45)	22.19 (18.88)	0.072
NRS leg	2.78 (2.34)	3.36 (2.67)	0.282
NRS back	3.05 (2.15)	4.15 (2.37)	**0.027**
EQ-5D	0.80 (0.22)	0.68 (0.28)	**0.022**
VAS health	77.10 (16.41)	71.79 (17.46)	0.150
Perceived recovery	6.05 (1.66)	4.94 (2.13)	**0.008**

One-year change			
ODI	−8.85 (6.73)	−11.89 (10.20)	0.111
NRS leg	−3.52 (2.70)	−3.79 (2.99)	0.671
NRS back	−2.95 (2.24)	−2.53 (2.81)	0.451
EQ-5D	0.25 (0.29)	0.27 (0.37)	0.788
VAS health	10.93 (22.07)	16.32 (23.88)	0.280

	n (%)	n (%)	

Patients achieving MCID			
ODI ≥11^19^	15 (37.5%)	29 (61.7%)	**0.024**
NRS leg ≥2^20^	31 (77.5%)	37 (78.7%)	0.891
NRS back ≥2^20^	29 (72.5%)	30 (63.8%)	0.388
EQ-5D ≥ 0.11^21^	23 (57.5%)	30 (63.8%)	0.546
VAS health ≥9.3^21^	21 (52.5%)	28 (59.6%)	0.507
*Perceived recovery ≥6^22^	33 (82.5%)	29 (61.7%)	**0.033**

In addition, the cohort was further subdivided according to HADS-Anxiety (HADS-A) and HADS-Depression (HADS-D) scores. Fifty patients had a normal HADS-A score at baseline, whereas 37 patients had an abnormal HADS-A score. The abnormal HADS-A group contained more females (p = 0.046), and had worse preoperative PROMS compared to the normal HADS-A group, but this was only significant for the ODI functionality (p = <0.001). In addition, one year postoperatively, patients with an abnormal HADS-A score at baseline scored worse on all outcome measures and this difference was significant for NRS back pain (p = 0.030) and perceived recovery scores (p = 0.024), but not for ODI (p = 0.074), NRS leg pain (p = 0.281), EQ-5D (p = 0.110) and VAS health (p = 0.341). For these patients, the one-year improvement in ODI, EQ-5D and VAS health scores was greater in the abnormal HADS-A group, while the change in NRS leg- and back pain was greater in the normal HADS-A group, but these differences were not significant. Finally, the proportion of patients achieving the MCID was comparable between both groups (Appendix A).

Fifty-four patients had a normal HADS-D score at baseline, whereas 33 patients had an abnormal HADS-D score. Baseline characteristics were comparable between both groups. Nevertheless, patients in the abnormal HADS-D group demonstrated worse preoperative PROMS compared with the normal HADS-D group, and these differences were significant for ODI, EQ-5D, and VAS health (p = <0.001 for all measures), but not for NRS leg pain (p = 0.068) and back pain (p = 0.076). At one year postoperatively, patients with abnormal baseline HADS-D scores reported poorer outcomes across all measures, except for NRS leg pain, and these differences were significant for ODI (p = 0.012), NRS back pain (p = 0.010) and EQ-5D (p = 0.013). Regarding postoperative improvement, greater changes in ODI, NRS leg pain, EQ-5D, and VAS health scores were observed in the abnormal HADS-D group, while improvements in NRS leg pain were greater in the normal HADS-D group. However, none of these differences were significant. Finally, the proportion of patients achieving the MCID was similar between both groups (Appendix B).

Significant Spearman correlations were observed between baseline HADS scores and preoperative ODI (ρ = 0.451, p = <0.001), EQ-5D (ρ = −0.355, p = <0.001) and VAS health scores (ρ = −0.325, p = 0.002). Moreover, significant correlations were present between baseline HADS scores and one year postoperative ODI (ρ = 0.269, p = 0.012), NRS back (ρ = 0.290, p = 0.006), EQ-5D (ρ = −0.334, p = 0.002), VAS health (ρ = −0.275, p = 0.010) and perceived recovery scores (ρ = −0.312, p = 0.003), thereby indicating that the preoperative HADS score can serve as a predictor for outcome. No significant associations were determined between baseline HADS scores and one-year change scores for all PROMS (Table 2).

**Table 2:** Spearman correlation coefficients for the association between HADS at baseline and preoperative, one-year postoperative and one-year change scores. Abbreviations: HADS; Hospital Anxiety and Depression Score, ODI; Oswestry Disability Index, NRS; Numeric Rating Scale, EQ-5D; EuroQol Five Dimensions Health Questionnaire, VAS; Visual Analogue Scale.

	*r*	*P*
Preoperative		
ODI (%)	0.451	**<0.001**
NRS leg	0.174	0.107
NRS back	0.110	0.313
EQ-5D	−0.355	**<0.001**
VAS health	−0.325	**0.002**

One year postoperative		
ODI (%)	0.269	**0.012**
NRS leg	0.003	0.980
NRS back	0.290	**0.006**
EQ-5D	−0.334	**0.002**
VAS health	−0.275	**0.010**
Perceived recovery	−0.312	**0.003**

One-year change		
ODI	−0.195	0.071
NRS leg	−0.062	0.566
NRS back	0.148	0.171
EQ-5D	0.079	0.469
VAS health	0.119	0.273

## 4 Discussion

This study demonstrates that patients with abnormal HADS scores at baseline have a worse baseline status with regard to leg pain, back pain, functional status, and quality of life. Furthermore, the study shows that postoperative outcomes after one year are poorer across all these parameters compared to patients with normal HADS scores. These findings are consistent with previous literature indicating that mental health status is a significant predictor of baseline symptom severity and postoperative outcomes in spinal surgery populations (Held et al., 2022; Carreon et al., 2020; Falavigna et al., 2015; Sinikallio et al., 2009; Derby et al., 2005).

It is noteworthy that the degree of improvement in clinical wellbeing in patients with abnormal HADS scores is comparable to the patients with normal HADS scores, with even a tendency to more improvement, including when the HADS-Anxiety and HADS-Depression subgroups were analyzed separately. This indicates that although patients with abnormal HADS scores had poorer absolute outcomes at one year, they experienced a similar degree of improvement following surgery. Furthermore, the proportion of patients achieving the MCID was largely comparable between the HADS groups, suggesting that an elevated preoperative HADS score does not necessarily preclude achieving a clinically meaningful improvement after surgery. The NRS leg pain scores were also similar between the groups both preoperatively and one year postoperatively, with only small differences observed in NRS back pain.

These findings suggest that PLIF surgery can produce meaningful functional improvements even in patients with elevated preoperative anxiety and depression. These findings are important in optimizing preoperative counseling for these patients. It helps to explain their poor baseline status and to lower their expectations on the postoperative outcome. Hopefully this will eventually lead to more patient satisfaction. The observed correlations between baseline HADS scores and both baseline parameters and one-year postoperative PROMs emphasize that baseline mental health status primarily affects symptom perception rather than the physiological response to surgery.

When analyzing the HADS subscales separately, patients with abnormal HADS-Depression scores reported significantly worse baseline ODI, EQ-5D, VAS health scores, whereas HADS-Anxiety scores were less consistently associated with preoperative PROMs. In agreement with that, patients with abnormal HADS-Depression scores demonstrated worse functioning and quality of life one year postoperatively, while this was not the case for the HADS-Anxiety group. This aligns with prior literature indicating that depression may have a stronger impact on functionality and quality of life after spine surgery compared with anxiety alone (Held et al., 2022; Sinikallio et al., 2011). This may be explained by anxiety being induced by the fear of the disease itself and for the treatment, while depression may be caused by a more intrinsic feeling of poor wellbeing. It is estimated that more than one third of spine surgery candidates demonstrate depressive symptoms preoperatively (Toivonen et al., 2021). A 2024 meta-analysis even estimated that the prevalence of depression is particularly high among patients undergoing spine surgery, with a prevalence of 46%, compared to 23% in the overall surgical population (Chen et al., 2024). These findings underscore the importance of comprehensive preoperative mental health assessment and suggest that in preoperative counseling, the focus should be on depression rather than on anxiety.

Taking mental status into account is important, as anxiety and depression may represent modifiable risk factors that can be addressed preoperatively. Temporal pharmacological treatment aimed at reducing preoperative anxiety and depression could be considered when clinically indicated, potentially in combination with psychological support or psychiatric consultation. Future studies are warranted to investigate whether targeted preoperative treatment of anxiety and depression can further improve outcomes following PLIF surgery.

## 5 Strengths and limitations

Several limitations of this study should be acknowledged. First of all, the relatively small sample size may have limited the statistical power to detect subtle associations. Additionally, although the HADS is a validated screening tool, it is not a diagnostic instrument, and underlying psychiatric disorders were not formally assessed. Furthermore, the HADS does not differentiate between state and trait anxiety. State anxiety refers to a transient emotional state characterized by feelings of tension and apprehensive cognition, whereas trait anxiety represents a stable personality trait reflecting individual differences in the likelihood to experience anxiety in response to stressful situations (Marteau and Bekker, 1992). In a surgical context, preoperative state anxiety is likely to be elevated due to the inherent stress of the perioperative period, whereas trait anxiety is by definition less context-dependent. As surgery is more likely to alleviate state anxiety than trait anxiety, and given that the HADS does not allow for differentiation between the two, the proportion of patients with true trait anxiety in our cohort remains uncertain. This may have led to an underestimation of the impact of preoperative anxiety on postoperative outcomes following PLIF surgery.

Another critique of the HADS is that, although it aims to assess the core symptoms of anxiety and depression while minimizing the influence of physical symptoms, some somatic aspects are nevertheless present in the questionnaire. One notable example is the item, “I can sit at ease and feel relaxed.” For individuals experiencing significant back and/or leg pain, sitting comfortably may be physically difficult regardless of their psychological state or ability to feel relaxed. Consequently, their response to this item may reflect physical limitations rather than symptoms of anxiety or depression. It is therefore important to recognize that, despite its intention to minimize the influence of physical symptoms, the HADS does not completely eliminate somatic factors from its assessment.

Finally, the study only evaluates outcomes at one year postoperatively, and longer-term follow-up is necessary to determine whether differences in PROMs persist beyond this period. Finally, it remains difficult to say whether depression results in more symptom burden or if increased symptom burden results in more depression. Therefore, addressing and improving both mental health and physical health is of great importance in providing the optimal care for this patient population. This study also has several notable strengths. The multicenter design enhances the generalizability of the findings across different patient populations and clinical settings. Additionally, the use of prospectively collected data represents a major strength, allowing for a reliable and temporal assessment of PROMs, while minimizing recall bias. Furthermore, focusing only on patients undergoing PLIF surgery may have reduced confounding, as different procedures can vary substantially in their indications, invasiveness and postoperative recovery trajectories, thereby increasing the internal validity of this study. Further research is warranted to evaluate whether interventions aimed at improving baseline HADS scores can positively influence postoperative outcomes, and to assess the cost-effectiveness of both pharmacological and non-pharmacological interventions in achieving these improvements.

## 6 Conclusion

Baseline anxiety and depression, as measured by the HADS score, are associated with worse preoperative clinical parameters. Patients with an abnormal HADS score at baseline show comparable improvements for all measures, but continue to report poorer quality of life, more pain, anxiety and depression and worse perceived recovery one year postoperatively compared with patients with normal baseline HADS scores. In preoperative counseling, these factors should be evaluated and discussed with the patients with a focus on depression rather than on anxiety. Addressing these factors preoperatively may facilitate more accurate expectation management, contributing to improved patient satisfaction.

## Authors’ contributions

CLAVL and LWFH formulated the research question. CLAVL, JK, GMO, WP and GCWR contributed to the development of the study design. LWFH performed the data analysis. LWFH prepared the first draft of the paper, with contributions and editing from CLAVL. All authors have read and approved the final manuscript.

## Ethics declaration

Written informed consent to take part in the study and to publish the article has been obtained from all participants or their legal representatives. The privacy rights of participants have been observed.

This study was performed in compliance with relevant laws, regulatory frameworks and guidelines where the research took place. This study was approved by the Medical Ethical Committee of Leiden The Hague Delft. (Approval No. NL83858.058.23)

The results of this clinical trial and any associated work have been posted in a registry. This clinical trial was registered with number NCT06361459 (ClinicalTrials.gov).

## Funding

No additional funding was received for this study. The subsidizing party for the original Smoking Cessation trial was the SGS-SAG fund Zilveren Kruis. The funders had no role in conceptualization, design, data collection, analysis, decision to publish or preparation of the manuscript. Peer review for the Smoking Cessation trial protocol was provided by the funding body.

## Conflict of interest

CLAVL: Thromboembolic complications in Neurosurgery (Covidien), Fundamentals of radiculopathy (YM Fund/Achmea health insurance and Eurospine), 5yr FU disc prosthesis in cervical spine surgery (CSRS E). Smoking in spondylodesis surgery (Achmea health insurance). Board of CSRS Europe, Netherlands Neurosurgical Society (NVvN) and Eurospine, Advisory Board for Rijndam Rehabilitation center, faculty for EANS, CSRS, Eurospine spine courses. LWFH, JK, GMO, WP and GCWR declare that they have no competing interests to disclose.

## Figures and Tables

**Table:** 

	HADS Category		
	Normal (HADS-A <8)	Abnormal (HADS-A ≥8)	P-value

N	50	37	
Male, n (%)	27 (54.0%)	12 (32.4%)	**0.046**
Smoker, n (%)	12 (24.0%)	8 (21.6%)	0.794

	Mean (SD)	Mean (SD)	

Age (years)	60.60 (10.10)	57.73 (9.28)	0.179
BMI	27.58 (3.84)	27.79 (4.62)	0.816
Preoperative			
ODI (%)	34.50 (15.35)	47.55 (16.95)	**<0.001**
NRS leg	6.52 (1.87)	7.08 (2.01)	0.183
NRS back	6.04 (2.10)	6.81 (2.05)	0.091
EQ-5D	0.52 (0.26)	0.41 (0.31)	0.083
VAS health	63.86 (17.32)	55.70 (23.03)	0.063
HADS-A	4.28 (2.06)	10.92 (2.97)	**<0.001**

One year postoperative			
ODI (%)	15.70 (18.66)	23.04 (18.71)	0.074
NRS leg	2.84 (2.38)	3.43 (2.70)	0.281
NRS back	3.18 (2.26)	4.27 (2.29)	**0.030**
EQ-5D	0.77 (0.25)	0.68 (0.27)	0.110
VAS health	75.74 (16.32)	72.19 (18.11)	0.341
Perceived recovery	5.88 (1.72)	4.86 (2.21)	**0.024**

One-year change			
ODI	−9.38 (7.52)	−12.00 (10.32)	0.174
NRS leg	−3.68 (2.68)	−3.65 (3.09)	0.960
NRS back	−2.86 (2.23)	−2.54 (2.97)	0.568
EQ-5D	0.26 (0.32)	0.27 (0.36)	0.813
VAS health	11.88 (23.37)	16.49 (22.75)	0.361

	n (%)	n (%)	

Patients achieving MCID			
ODI ≥11 (Lauridsen et al., 2006)	21 (42.0%)	23 (62.2%)	0.063
NRS leg ≥2 (Farrar et al., 2001)	40 (80.0%)	28 (75.7%)	0.629
NRS back ≥2 (Farrar et al., 2001)	35 (70.0%)	24 (64.9%)	0.612
EQ-5D ≥ 0.11 (Cheng et al., 2024)	30 (60.0%)	23 (62.2%)	0.838
VAS health ≥9.3 (Cheng et al., 2024)	27 (54.0%)	22 (59.5%)	0.612
*Perceived recovery ≥6 (van Grafhorst et al., 2024)	39 (78.0%)	23 (62.2%)	0.107

**Table:** 

	HADS Category		
	Normal (HADS-D <8)	Abnormal (HADS-D ≥8)	P-value

N	54	33	
Male, n (%)	22 (40.7%)	17 (51.5%)	0.327
Smoker, n (%)	9 (16.7%)	11 (33.3%)	0.073

	Mean (SD)	Mean (SD)	

Age (years)	59.41 (10.23)	59.33 (9.23)	0.973
BMI	27.24 (4.13)	28.37 (4.20)	0.224
Preoperative			
ODI (%)	34.53 (15.71)	49.09 (15.71)	**<0.001**
NRS leg	6.46 (1.91)	7.24 (1.90)	0.068
NRS back	6.06 (2.18)	6.88 (1.88)	0.076
EQ-5D	0.55 (0.26)	0.34 (0.29)	**<0.001**
VAS health	66.63 (16.79)	50.18 (21.45)	**<0.001**
HADS-D	4.44 (1.68)	11.00 (2.67)	**<0.001**

One year postoperative			
ODI (%)	14.66 (16.68)	25.63 (20.62)	**0.012**
NRS leg	3.13 (2.56)	3.03 (2.51)	0.860
NRS back	3.15 (2.19)	4.45 (2.33)	**0.010**
EQ-5D	0.79 (0.23)	0.65 (0.29)	**0.013**
VAS health	76.87 (16.13)	69.91 (17.99)	0.065
Perceived recovery	5.54 (2.13)	5.30 (1.78)	0.599

One-year change			
ODI	−9.91 (7.00)	−11.45 (11.32)	0.483
NRS leg	−3.33 (2.88)	−4.21 (2.75)	0.164
NRS back	−2.91 (2.48)	−2.42 (2.71)	0.396
EQ-5D	0.24 (0.30)	0.31 (0.38)	0.336
VAS health	10.24 (21.28)	19.73 (25.00)	0.063

	n (%)	n (%)	

Patients achieving MCID			
ODI ≥11 (Lauridsen et al., 2006)	25 (46.3%)	19 (57.6%)	0.307
NRS leg ≥2 (Farrar et al., 2001)	41 (75.9%)	27 (81.8%)	0.519
NRS back ≥2 (Farrar et al., 2001)	39 (72.2%)	20 (60.6%)	0.260
EQ-5D ≥ 0.11 (Cheng et al., 2024)	29 (53.7%)	24 (72.7%)	0.078
VAS health ≥9.3 (Cheng et al., 2024)	28 (51.9%)	21 (63.6%)	0.282
*Perceived recovery ≥6 (van Grafhorst et al., 2024)	40 (74.1%)	22 (66.7%)	0.459

## List of abbreviations

PLIF: Posterior Lumbar Interbody Fusion; HADS: Hospital Anxiety and Depression Scale; PROMS: Patient Reported Outcome Measures; ODI: Oswestry Disability Index; NRS: Numeric Rating Scale;

EQ-5D: Five Dimensions Health Questionnaire; VAS: Visual Analogue Scale; BMI: Body Mass Index; MCID: Minimal Clinically Important Difference; HADS-A: HADS-Anxiety; HADS-D: HADS-Depression.

## Appendices

### Appendix A

Appendix A. Overview of study participants demographic data, differentiated by the HADS-Anxiety score. Abbreviations: HADS-A; Hospital Anxiety Score, BMI; Body Mass Index, ODI; Oswestry Disability Index, NRS; Numeric Rating Scale, EQ-5D; EuroQol Five Dimensions Health Questionnaire, VAS; Visual Analogue Scale, MCID; Minimal Clinically Important Difference. *Perceived recovery assessed one year postoperatively.

### Appendix B

Appendix B Overview of study participants demographic data, differentiated by the HADS-Depression score. Abbreviations: HADS-D; Hospital Depression Score, BMI; Body Mass Index, ODI; Oswestry Disability Index, NRS; Numeric Rating Scale, EQ-5D; EuroQol Five Dimensions Health Questionnaire, VAS; Visual Analogue Scale, MCID; Minimal Clinically Important Difference. *Perceived recovery assessed one year postoperatively.
